# Going beyond electrospray: mass spectrometric studies of chemical reactions in and on liquids

**DOI:** 10.1039/c5sc02740c

**Published:** 2015-10-01

**Authors:** Andrew J. Ingram, Cornelia L. Boeser, Richard N. Zare

**Affiliations:** a Department of Chemistry , Stanford University , Stanford , CA 94305 , USA . Email: zare@stanford.edu; b Thermo Fisher Scientific , San José , CA 95134 , USA

## Abstract

There has been a burst in the number and variety of available ionization techniques to use mass spectrometry to monitor chemical reactions in and on liquids. Chemists have gained the capability to access chemistry at unprecedented timescales, and monitor reactions and detect intermediates under almost any set of conditions. Herein, recently developed ionization techniques that facilitate mechanistic studies of chemical processes are reviewed. This is followed by a discussion of our perspective on the judicious application of these and similar techniques in order to study reaction mechanisms.

## Abstract 2

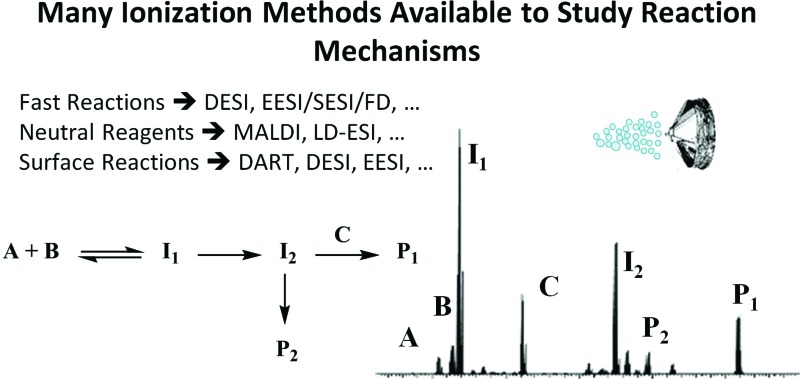
Recently developed and applied ionization techniques have brought mass spectrometry to bear on previously inaccessible chemistry. We offer our perspective on this field and its application for studying reaction mechanisms.

## 1. Introduction

The vast majority of reactions of interest to synthetic chemists occur in liquids. Mechanistic studies of these processes tend to rely on the creativity and experience of chemists to envision plausible intermediates and reasonable reaction pathways. However, unanticipated species and novel mechanisms can be easily overlooked. Furthermore, transient intermediates that form during reactions can often be difficult, or even impossible, to isolate, making them extremely challenging to study. Mass spectrometry (MS) complements other spectroscopic techniques (*e.g.* UV/vis, IR, and NMR) in analyzing reactions occurring in solution. MS monitoring of chemical reactions is a powerful tool to directly track reaction progress, detect and study reactive compounds, and interrogate mechanisms through cleverly designed experiments. An important advantage of mass spectrometry for studying reactions, which are generally complicated mixtures containing species with vastly different concentrations, is that some mass spectrometers can simultaneously detect gas-phase ions of astonishingly disparate relative abundance. Readily available commercial instruments can have intraspectral dynamic ranges greater than 10^5^.^1^

There has been a significant advance in mass spectrometry, known as ambient ionization which is defined as “ionization at atmospheric pressure with little or no sample preparation”.^2^ Electrospray ionization (ESI), initially developed by Dole and coworkers in 1968 (ref. 3) and, as realized by Fenn in the 1980's,^4^ was in some sense the first ambient ionization technique. It operates by spraying a plume of charged droplets from an analyte solution at atmospheric pressure. These droplets are drawn into the heated inlet of a mass spectrometer where they evaporate and release ionized compounds for detection. ESI has a number of characteristics that have made it the method of choice for solution MS reaction monitoring, providing the solvent is conductive.^5^ A few prominent attributes are the following: first, it is well suited to ionize mixtures of compounds dissolved in homogeneous solutions, the most common phase for synthetic chemistry; however, these data are generally qualitative. Second, electrospray provides a constant, and not pulsed, source of ions which lends itself to time-resolved studies of reactivity from seconds to minutes. Third, ESI is a “soft” ionization technique, where detected analytes are not generally fragmented. Finally, because it is a ubiquitous technology, most researchers have access to the requisite instrumentation.

On the other hand, there are significant limitations of ESI as an analytical technique for monitoring chemical processes: (i) reaction times of less than a few seconds are very difficult to achieve without sophisticated instrumentation; (ii) it is not amenable to ionization from nonpolar, nonconductive solvents except under very special circumstances; and (iii) compounds generally must be charged or contain acidic/basic functionalities in order to be readily ionized. Therefore, it is beneficial to look beyond ESI for new techniques to observe condensed-phase reactions in real time. In this perspective, we review recently developed ionization methods that have been applied to monitor reactions and allow unique access to molecular reactivity that was not previously realized. We also discuss promising areas primed for further development as well as our perspective on the general use of MS to effectively study mechanism and reaction intermediates.

There has been a revolution in the development of new, ambient ionization methods specifically designed to analyze complex samples in varied environments with no sample preparation.^2^ These developments have granted new flexibility to mass spectrometrists to directly couple samples to MS. For the reaction chemist, these techniques can give access to unprecedented reaction timescales and enable ionization and detection of intermediates under virtually any condition. Rather than alter a process to allow for ESI-MS monitoring, the flexibility and variety of available techniques now allows for direct coupling of reactions to mass spectrometry. In essence, the modern chemist has the ability to choose, or even develop, an ideal method to sample a particular reaction in terms of timescales, sensitivity, and compatible ionization characteristics.

In the simplest implementation of reaction monitoring by MS, known as an “offline” analysis, researchers typically take aliquots of ongoing reactions, dilute them to concentrations amenable for ESI-MS, and infuse these solutions into an electrospray source. Aliquots are sometimes quenched chemically or thermally to prevent species from degrading prior to analysis. Additional reagents can be added to assist or modify the ionization process. In contrast to these “offline” methods, “online” monitoring of chemical reactions involves interfacing a reactor with an ionization source so that the reaction is directly analyzed in real time. A wide variety of tools have been developed for online ESI experiments,^5^ including microreactors to initiate reactions in the tubing leading to the ion source. Microreactors allow moderately fast reactions to be analyzed (milliseconds up to approx. 3–30 seconds).^6^ There are also methods to interface conventional reaction flasks to ESI sources that allow for continuous monitoring by ESI-MS.^7^ Importantly, these approaches allow for simultaneous acquisition of data when combined with other techniques that are able to interface with the same reaction flask (*e.g.*, aliquot sampling for GC, HPLC, or NMR, or *in situ* UV/vis or IR). Atmospheric pressure chemical ionization (APCI) has also been an important method for reaction monitoring and some mechanistic studies.^8^

In the more than two decades since the original applications of ESI-MS to study mechanisms in reacting solutions,^5^ many studies have been published that describe its applications to solution chemistry. An excellent monograph on the field, edited by Santos, was published in 2010.^5^ Furthermore, a significant number of excellent reviews and book chapters have appeared recently, covering various aspects of this topic, including general reaction monitoring with ESI,^9–11^ reactions occurring in ESI microdroplets,^12,13^ applications to biological macromolecules,^6,14^ electrochemical MS,^15^ cold-spray ionization for thermally unstable compounds,^16^ and gas-phase ion spectroscopy of reaction intermediates.^17,18^ There are a few recent reviews of time-resolved mass spectrometry,^19^ instrumentation used for reaction studies,^20^ general monitoring of chemical transformations with MS,^21^ and the application of ambient ionization methods to study interfacial ion–molecule reactions^22^ that incorporate some or most of the methods mentioned herein.

In this perspective, we survey the field of direct MS reaction monitoring that use ionization methods other than ESI or APCI, with an emphasis on solution-phase mechanistic studies. Because there is little practical difference between monitoring a reaction to detect intermediates *versus* determining progress, we will discuss reaction progress monitoring and “offline” techniques/studies where appropriate. Our hope is that this perspective can serve to bridge the fields of ambient ionization, mass spectrometry, chemical catalysis, organic chemistry, and physical mechanistic chemistry. Electrochemistry mass spectrometry^15^ and time-resolved mass spectrometry of biochemistry^6^ are very active fields in reaction monitoring that rely on the development of new ionization techniques; however, they have both recently been very well reviewed and the interested reader is directed to the publications mentioned above and references therein. We believe that the advent and implementation of these new ionization techniques provides previously unrealizable flexibility in designing experiments to explore reaction mechanisms and discover new chemistry.

## 2. Methodology

### 2.1 Liquid phase reactions

#### 2.1.1 Electrospray-based ionization

##### 2.1.1.1 Single source techniques

###### Desorption electrospray ionization

**Fig. 1:**
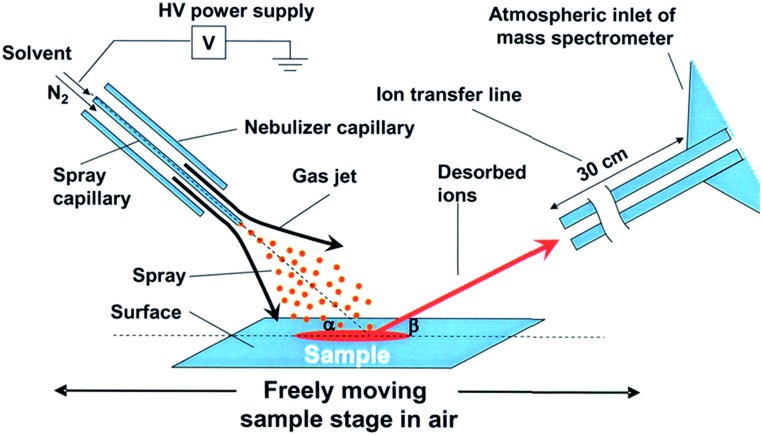
Diagram of DESI process. From ref. 23. Reprinted with permission from AAAS.

Desorption electrospray ionization (DESI) is a surface ionization variant of ESI developed in 2004 (ref. 23) that has become widely used in a variety of fields (Fig. 1). Applications include forensics, homeland security, pharmaceutical applications, and tissue imaging. A DESI source is very similar to ESI in that it consists of coaxial outer and inner capillaries. The inner capillary carries pure solvent or a reagent solution to the spray tip, and the outer capillary carries high-pressure nebulizing gas to generate a plume of droplets. An ionizing potential can be applied at the source or to the solution just prior to entering the source. Charged solvent droplets are sprayed onto a surface that is impregnated with analyte, such as a catalyst or an enzyme. As the droplets hit the surface, the imbedded analytes are extracted into an incipient liquid film. Secondary microdroplets containing the extracted analyte are generated by the splash of primary droplets from the source and intercepted by the mass analyzer. This relatively simple setup is modular in that ionizing solvents and reagent sprays can be readily varied without affecting sample preparations of the surface.

The transition of DESI from a purely analytical technique to a system for initiating and studying reactions at short timescales is worth discussing. An early modification of DESI was to add derivatizing reagents to the spray in order to facilitate analysis of compounds on the surface. The Cooks group initially tested the use of spray additives to enhance the signal of explosives on various surfaces, where the addition of hydrochloric acid into the spray solvent led to increased formation of Cl^–^ adducts in negative ion mode.^24^ This concept of “reactive DESI” was later expanded to derivatization of analytes on the surface by sprays containing analyte-specific reagents.^25^ *cis*-Diol functionalities in carbohydrates were selectively recognized *via* cyclization with phenylboronate anions added to the DESI spray. Chemical derivatization in the DESI spray was quickly applied to study chemical reactions. Early work in this area was published by Sparrapan *et al.*,^26^ where they used DESI to investigate the Eberlin reaction, the acetylation or *trans*(acetylation) of carbonyls in the gas phase.

**Fig. 2:**
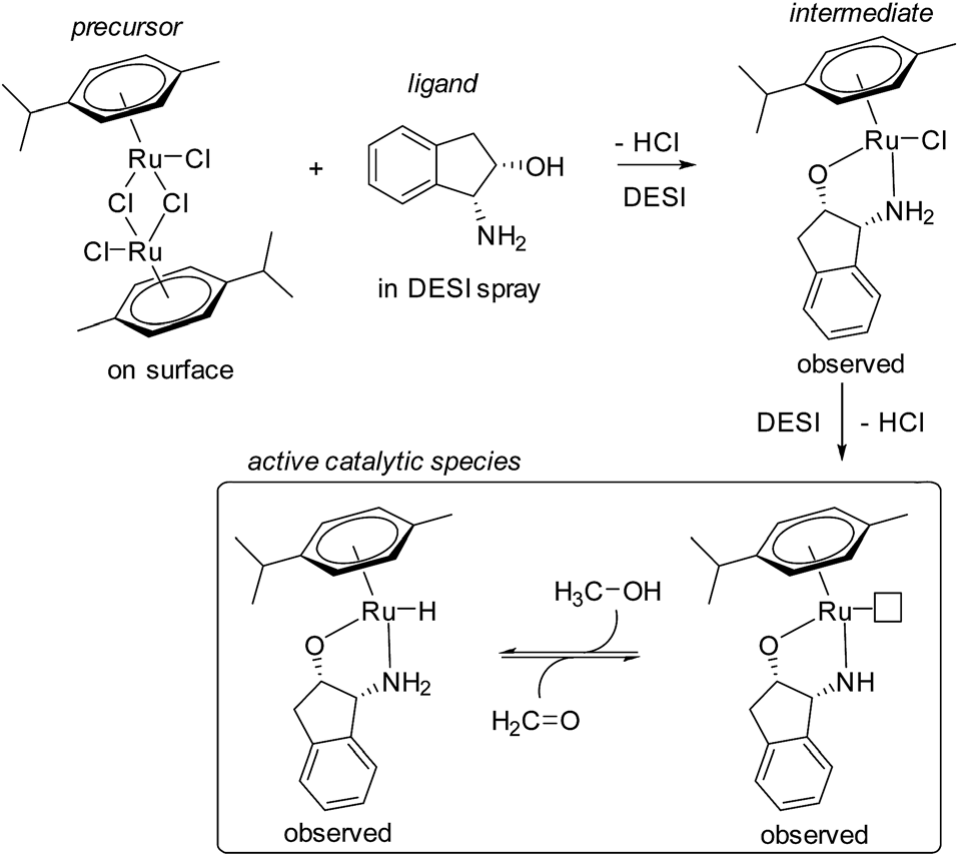
Proposed mechanism for the formation of a Ru(ii) transfer hydrogenation catalyst observed by DESI-MS.

Perry *et al.* were the first to apply DESI to simultaneously initiate reactions on surfaces and observe intermediates within milliseconds of droplets leaving the surface.^27^ The simultaneous synthesis and function of a Ru(ii) transfer hydrogenation catalyst was investigated (Fig. 2), where the ruthenium precursor was deposited onto paper and sprayed with the ligand dissolved in methanol. Signals corresponding to the protonated versions of precatalyst and various forms of the catalyst were identified. Subsequent work on this system investigated more methanol oxidation intermediates and catalyst degradation products.^28^

A number of publications quickly followed that described liquid-phase reactions analyzed by DESI-MS.^28–32^ At the same time as the initial publication by Perry *et al.*,^27^ Xu *et al.* used DESI to study the Eschweiler–Clarke reaction, where amines are methylated using excess formaldehyde and formic acid.^30^ Two key intermediates were detected: the nucleophilic addition product of the amine with formaldehyde, and an iminium ion that results from dehydration of that initial addition product. Both have such short lifetimes in acidic media that they could not be detected by conventional ESI. Zhang *et al.* used DESI to detect products of Schiff base reactions between various amines sprayed with acetone.^31^ Chung *et al.* used DESI to detect intermediates of a chemoselective Pd-catalyzed oxidation of vicinal diols, where masses corresponding to a catalytically important Pd–diol chelate were observed.^32^

Because reaction and analysis times during DESI are on the order of milliseconds or less, the method is well suited to detect short-lived intermediates in solution. Perry *et al.* investigated catalytic intermediates in an oxidative C–H amination reaction catalyzed by a dirhodium complex.^29^ Based on DFT calculations, a mechanism had been proposed suggesting a Rh-nitrene with a microsecond lifespan as a key N-transfer intermediate. Using DESI, a surface containing the rhodium catalyst and substrate was sprayed with a solution containing a nitrene source. The postulated Rh-nitrene intermediate was detected as a Na^+^-adduct. Using a deuterated solvent (dichloromethane), the investigators found that, in the absence of suitable concentrations of substrate, the nitrene abstracts a hydrogen atom from the solvent. This generates a mixed valent Rh(ii/iii) dimer that is believed to be inactive. Similar to this Rh chemistry, Gouré *et al.* also detected a short-lived diiron(iii/iv) imido intermediate that was active for nitrene transfer and amine synthesis.^33^ Again, abstraction of a hydrogen atom from solvent competed with productive nitrene transfer.

DESI is also useful for the identification of catalyst decomposition mechanisms. Flender *et al.* determined that the C–H hydroxylation catalyst [(Me_3_tacn)RuCl_3_] as well as its tribromide equivalent undergo dimerization during catalysis (Me_3_tacn = 1,4,7-trimethyl-1,4,7-triazacyclononane).^34^ This process was shown to be a deactivation pathway because adding more substrate did not lead to more product formation. In a separate study utilizing a combined approach of DESI-MS, time-resolved sonic-spray ionization (SSI), and vibrational spectroscopy, Ingram *et al.* examined the activation mechanism of a Cp*Ir precatalyst for catalytic water oxidation with periodate as the terminal oxidant (Cp* = pentamethylcyclopentadienyl). They determined that nonselective hydroxylation and oxidation of the Cp* ligand by the periodate oxidant was occurring, which leads to complete degradation of the Cp* ring into CO_2_ and organic acid byproducts.^35^

In terms of recent electrochemistry-MS developments, Brown *et al.* applied DESI to sample intermediates electrochemically generated directly from an electrode surface.^36^ Analytes were sprayed from the DESI source and onto a wetted Pt-working electrode. This electrode was on the outside edge of a wheel, partially submerged in a bath of solvent, and the DESI spray was directed at the top of the exposed section of the wheel. To maintain electrical contact, the liquid film on the electrode was constantly replenished by rotating the wheel during analysis. The fast sampling times of DESI allowed for the detection of highly reactive electrochemical intermediates. Previously undetected intermediates of uric acid and xanthene oxidations were identified. In subsequent work on the oxidative dimerization of *N*,*N*-dimethylaniline, an incredibly unstable singly oxidized radical cation was readily observed.^37^ This radical cation can be generated in small amounts during the ionization process, however, the authors found its intensity to significantly increase when a potential was applied to the working electrode. This work showed that DESI is capable of sampling intermediates from surfaces that react with diffusion limited rate constants, which suggested that the delay between surface sampling and ionization can be on the order of microseconds.

The majority of these studies exploited the characteristics of DESI to examine specific aspects of chemistry. The very short timescales of DESI makes it ideal to study highly reactive intermediates that are prepared “on-the-fly” during the ionization process, or sampled from a surface.^29,33,37^ Furthermore, catalyst activation mechanisms can be specifically targeted.^27,35^ Perhaps even more important than the timescale of DESI is the simplicity of the technique. In practice, optimizing concentrations and screening reagents during ESI-MS studies of reactions is tedious. Changing from one reagent to another often requires disassembly and extensive cleaning of the microfluidics equipment to avoid cross contamination. During DESI, surface reagents and concentrations can be varied freely without noticeable carryover. This allows for much more rapid screening of reagents and reaction conditions in the search for particular intermediates. One significant drawback of DESI is that it is not generally suitable for examining air-sensitive chemistry; however, in some cases atmospheric degradation can be mitigated or avoided.^28,35^

###### Ionization methods without an applied voltage

In some cases, the ionizing potential of ESI and related techniques can lead to artifactual species, generated by direct oxidation or reduction of analytes in the source.^38^ These compounds can be particularly deleterious to mechanistic studies of reaction intermediates because the origin of each observed ion should be clear. It should be noted that the electrochemistry DESI experiments discussed above were conducted without an ionizing potential applied to the ion source. Instead, ions were generated at the electrode surface and transported to the mass spectrometer in the DESI microdroplets.

Sonic-spray ionization (SSI) is a variant of ESI where ions are generated without the application of a high voltage to the analyte solution. Instead, very high gas flows cause nebulization of the analyte solution into very small microdroplets which are slightly charged due to stochastic variations in total charge. Subsequent ionization of dissolved analytes occurs through a mechanism similar to ESI.^39^ In the combined DESI/SSI study mentioned above, Ingram *et al.* interfaced an online flow reactor with SSI to monitor the oxidative activation of a homogeneous organometallic Ir water oxidation catalyst.^35^ The online reactor system resolved 3–14 second reaction times. In this case, SSI was used because the applied voltage in ESI induced oxidative degradation of the Ir catalyst, even in the absence of an external oxidant. Thus, SSI allowed this system to be studied in absence of such complicating instrumental artifacts.

Easy ambient spray ionization (EASI) is a DESI variant in which no high voltage is applied to the analyte solution, making EASI similar to SSI.^40^ EASI has been applied to monitor the polymerization of a siloxane surface coating *in situ*, a mixed phase surface process as well as used in a number of other analytical applications.

Perhaps one of the simplest approaches to MS reaction monitoring was developed in the Chen laboratory (National Chiao Tung University). By placing one end of a fused silica capillary into a sealed reaction flask with a slight over pressure and putting the other end in front of an MS inlet, Hsieh *et al.* were able to detect ions from solution and monitor the deesterification of a protected sugar.^41^ The over pressure induced flow through the tubing in the range of nL min^–1^. In this case, the voltage at the MS inlet was believed to induce ionization. While the utility of this technique is not clear and there were problems in terms of signal stability, dead times, and sensitivity, its simplicity is most impressive.

###### Nanospray ionization with theta-glass capillaries

**Fig. 3:**
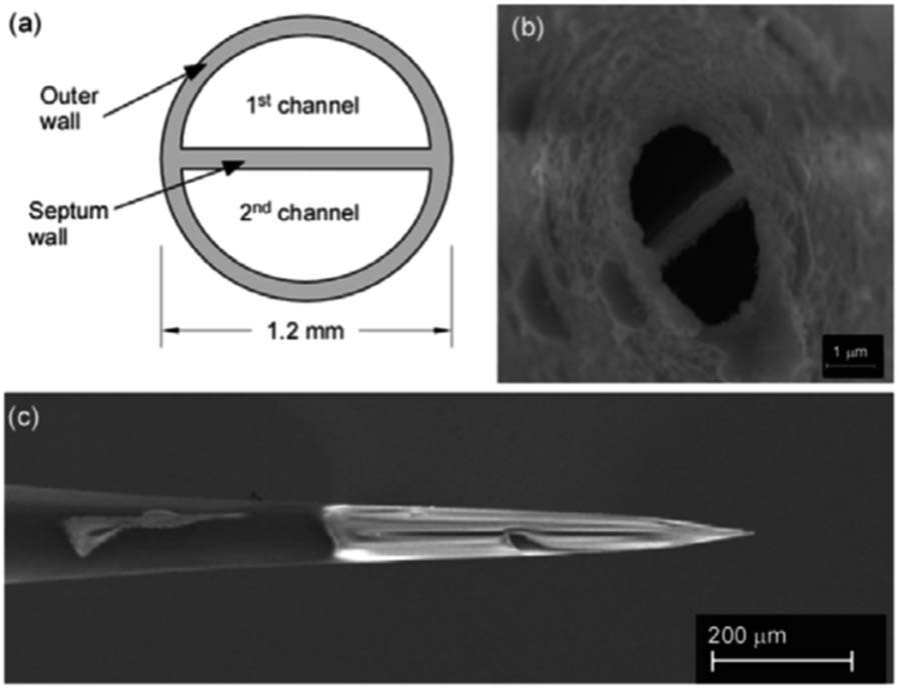
Theta-shaped profile of glass capillary (a) sketch of the distal end and (b) electron micrograph of the very tip of a sputter-coated emitter and (c) electron micrograph of the side of the emitter showing hand-painted platinum-coating to the left of the tip. Copyright 2012 IM Publications. Reproduced with permission from ref. 42.

Nanospray ionization through theta-glass capillaries is a recently developed technique.^42^ “Theta” capillaries are round glass capillaries with two channels separated by a glass wall divider, thus appearing to form the Greek letter *Θ* when viewed head on (Fig. 3). The technique involves the mixing of two solutions at the tip of a theta capillary prior to droplet formation and ionization. This mixing is likely similar to an early dual channel ESI technique, where two reagent flows were mixed at the Taylor cone of a microchip ESI emitter.^43^ As with regular nanospray emitters, the theta-glass capillaries are pulled with high precision instruments to form micrometer-sized tips and can generate a range of droplet sizes similar to nanospray. Because nanospray ionization often generates droplets with microsecond lifetimes, theta-glass capillaries provide the opportunity to initiate and monitor reactions within microseconds, perhaps representing one of the current limits for low timescale reaction studies with techniques based on electrospray.

Theta-glass capillaries have been applied to a few reaction studies, including initial work studying H/D exchange reactions and formation of noncovalent complexes.^42^ Very recently, a well-designed study was performed to estimate mixing times and droplet lifetimes during electrospray from theta-glass capillaries.^44^ First, it was shown that efficient droplet mixing occurred by mixing a solution of K^+^ with one of Na^+^ and 18-crown-6 ([18C6]), and monitoring the exchange of Na^+^ for K^+^. In all cases, the data agreed with the predicted equilibrium ratios of [K·18C6]/[Na·18C6] as well as the data obtained from premixing the solutions, suggesting that mixing is complete prior to complete ionization. Models assuming purely diffusion-based mixing in droplets estimated maximum mixing times of tens to hundreds of microseconds. However, models that incorporate turbulence suggest times of under a microsecond. These estimates did not include the degree of mixing that occurs at the tip of the emitter prior to droplet formation.

It was possible to estimate droplet lifetimes by monitoring a well-characterized reaction, the reduction of 2,6-dichloroindophenol by l-ascorbic acid. The product-to-reactant ratio measured by MS suggested the lifetimes were on the order of tens of microseconds, incorporating rough estimates for reaction rate enhancement due to droplet evaporation. Given that nanospray droplet sizes and lifetimes highly depend on a variety of factors, including an emitter's outer diameter and the ionizing potential, simple adjustments to the emitter could yield significantly different results.^45^ This presents both an opportunity and challenge for future reaction studies using theta-glass capillaries.

###### Ultrasonication-assisted spray ionization (UASI)

Ultrasonication has been shown to significantly enhance reaction rates in solution, as well as induce flow through a silica capillary placed under the surface of a solution. If the opposite end of the capillary is held in front of the inlet of a mass spectrometer, ions corresponding to analytes in the ultrasonicated solution can be observed.^46^ Acoustic waves are believed to cause solution nebulization into microdroplets at the end of the capillary, and subsequent ionization occurs by a mechanism similar to ESI. This ionization technique was shown to be effective for online reaction monitoring, where the ultrasonication accelerated deesterification of protected sugars with NaOMe were analyzed.^46^ While this technique is not broadly applicable to mechanistic studies of reactions performed without sonication, it is a brilliant example of a nonelectrospray technique bringing MS to bear on a reaction *in situ*. It is difficult to imagine interfacing conventional NMR spectroscopy to a reaction in an ultrasonication bath.

###### Droplet spray ionization from glass microscope slides

There is a significant limitation to analyses of reactions initiated within the tubing leading to an ESI source, where the reacting solution must travel the length of the capillary prior to ionization and analysis. This leads to a delay between the start of a reaction and when that reaction can be analysed (*i.e.* a deadtime). Furthermore, monitoring reactions over different time periods requires physically shortening the length of tubing between the ESI source and the initiation site, which can introduce variability and potentially a lack of repeatability into an experiment. In part to circumvent these issues, a technique, ambiguously named droplet spray ionization, was developed.^47^ Applying a high voltage to liquids deposited onto the corner of a glass microscope slide placed in front of a mass spectrometer inlet induces electrospray at the corner of the slide. By sequentially adding reagents to an already spraying system, it is possible to observe reactions with very short deadtimes on the order of solution mixing and ESI ionization. This technique was applied to monitor the activation and ethylene polymerization behaviour of dicyclopentadienyl-zirconium(iv) dichloride (zirconocene dichloride)/methyl-aluminoxane (MAO) cocatalyst mixtures.^47^

###### Electrostatic-spray ionization mass spectrometry

Electrostatic-spray ionization (ESTASI) was developed as a tool to induce ionization from liquids without having the analyte solution come in contact with a high voltage electrode. By separating the high voltage electrode and the analyte solution with an insulating layer, such as a micropipette tip or a layer of poly(methylmethacrylate), it is possible to induce spray and ionization from pipette tips or small droplets.^48^ This technique is very well suited to online analysis of droplets in microfluidic channels as an electrode can be placed on one side of a chip, while the mass spectrometer can be placed on the other. A small, 50 μm, “spyhole” cut into the surface of the channel opposite from the electrode allows the spray from an aqueous droplet to reach the spectrometer. This technique has been used to monitor a microdroplet-based tryptic digestion as well as a biphasic reaction between β-lactoglobulin and α-tocopheryl acetate.^49^

##### 2.1.1.2 Dual-source techniques

This section discusses techniques that utilize two independent flows or sprays of solvent/reagent to carry analytes or initiate reactions on the way to the instrument. In some cases, one flow consists of analyte while the other carries charge for ionization of analytes in the other flow. In other cases, intermediates are generated in flight by introducing reagents in each of the flows such that reactions initiate during ionization.

###### Alphabet soup

There has been some discussion in the literature regarding the large number of acronyms introduced for highly related dual-source ambient ionization techniques.^50^ In particular, controversy surrounds a number of techniques which have been applied to monitor reactions and detect intermediates.^51,52^ It has been suggested that different acronyms should be used for gas-phase *versus* solution-phase ionization.^51^ In the context of this review, we have approached this problem by organizing the articles in the literature by the reactivity intended to be studied rather than stated techniques. We have noted when a given technique was specified by the authors in the original publications. We should point out that even these divisions are somewhat arbitrary as it is not always clear, regardless of authors' intentions, whether a given compound was formed in solution, at an interface, in the gas phase, or some combination of all three.

###### Extractive electrospray, secondary electrospray, and fused droplet ionization

**Fig. 4:**
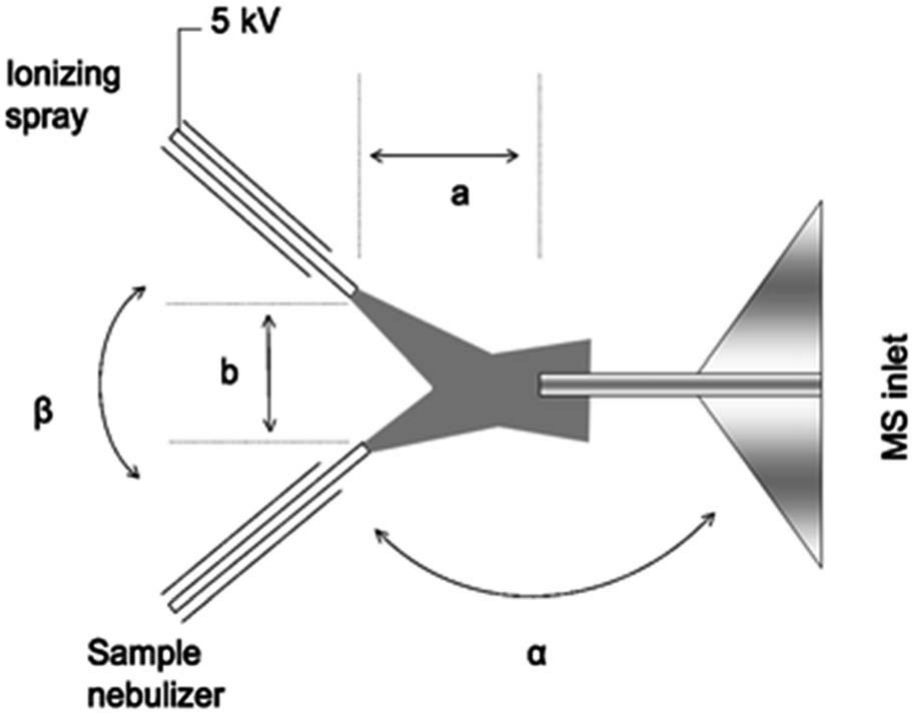
Schematic diagram of extractive electrospray ionization showing ionizing and sample sprays. Reproduced from ref. 53 with permission of the Royal Society of Chemistry.

Extractive Electrospray Ionization (EESI) was first reported in 2006 by Chen *et al.* as a method to directly analyze complex liquid samples without dilution or pretreatment.^53^ The original technique consisted of two nebulizing spray sources: one was an uncharged nebulizer containing the sample to be analyzed, while the other was a conventional ESI source which sprayed charged droplets of pure solvent (see Fig. 4). The sprayers were oriented such that the two plumes of droplets crossed a few millimeters from the sprayer tips. Droplets collided, and analytes in the sample became ionized from the charge supplied by droplets in the ESI plume. The precise details of the ionization mechanism of EESI have not been fully elucidated. However, the picture that has emerged^54^ is that droplets from each plume collide and then fragment apart after sharing material. Analytes transferred from the sample droplets into the charged droplets are ionized in a similar manner to ESI.

EESI is very similar or perhaps identical to two other dual-spray techniques that use nearly identical equipment and setups: secondary electrospray ionization (SESI), developed by Wu and coworkers in 2000;^55^ and fused-droplet electrospray ionization (FD-ESI), developed by Chang and coworkers in 2002.^56^ Some have made a distinction between SESI and EESI by stating that ionization occurs in the gas phase in SESI and in droplet phase in EESI;^50–52^ however, these phenomena are not necessarily exclusive and it can be difficult to make this distinction in practice.

EESI, SESI, and FD-MS have been applied in a number of online reaction monitoring experiments. Zhu *et al.* developed a system for monitoring reactions with volatile reagents where the headspace of a reaction flask was constantly purged with N_2_(g) and the exhaust was used as the sample “spray” for SESI ionization.^57^ They monitored the Michael addition of phenylethylamine to acrylonitrile and the *N*,*N*-dimethyl-4-aminopyridine (DMAP)-catalyzed acetylation of benzyl alcohol with acetic acid. In the latter reaction, they were even able to observe what were proposed to be DMAP-bound intermediates. This very simple approach to online reaction monitoring could be readily implemented for any reaction with volatile reagents. Furthermore, reactions with conditions not generally amenable to ESI could be studied, such as those with nonpolar solvents, high concentrations of reagents or salts, and so on.

**Fig. 5:**
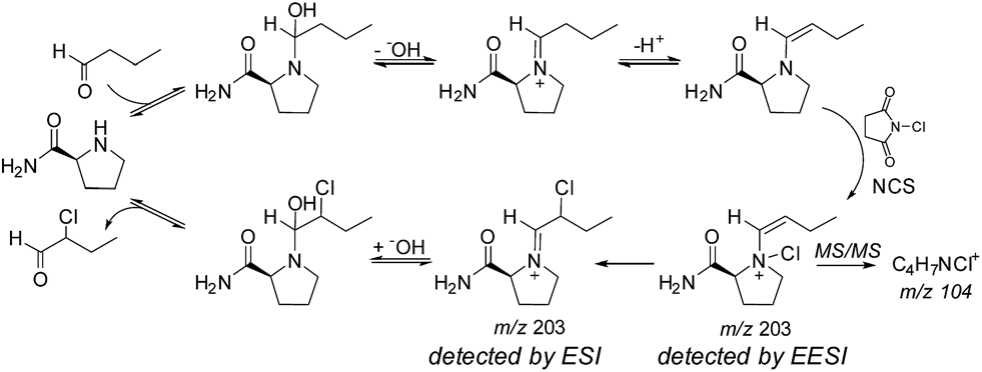
Mechanism of l-prolineamide catalyzed aldehyde α-chlorination. Copyright 2007 Wiley-VCH Verlag GmbH & Co. KGaA, Weinheim. Adapted with permission from ref. 58.

In perhaps the first application of an ambient ionization technique to initiate and study solution phase reactions during an analysis, Marquez *et al.* used EESI to probe for intermediates present during the l-prolineamide catalyzed α-halogenation of aldehydes with *N*-chlorosuccinimide (NCS).^58^ After studying the ongoing reaction with a variety of ESI techniques and identifying a number of species, they used a dual-spray setup to study the reactions at millisecond timescales in the hopes of detecting fleeting intermediates. In this publication, they referred to the technique as dual-ESI. By infusing a solution of butanal and l-prolineamide in one source while infusing a solution of NCS in the other, Marquez *et al.* detected intermediates and products formed by reactions that had initiated upon droplet/droplet collisions and were analyzed within milliseconds. These studies revealed the formation of a new species, proposed after a series of tandem MS (MS/MS) investigations to be an important *N*-chlorinated adduct of an enamine intermediate (see Fig. 5). This same group also applied EESI to detect a proposed distonic radical cation present during the electron-transfer-catalyzed dimerization of *trans*-anethole.^59^

Yao *et al.* creatively used FD-MS to safely study the reactivity of a phenoxathiin radical with a variety of organic compounds.^60^ Phenoxathiin radicals are generally isolated as potentially explosive perchlorate salts. In this study, the investigators used the electrochemistry of the ESI source to generate the phenoxathiin radical directly before analysis within the ESI plume. By introducing another reagent in the second spray, they were able to screen the reactivity of this radical with a large variety of organic compounds without having to isolate this reactive compound. While it is not entirely clear whether reactions occurred within the evaporating microdroplets or within the gas phase, this study highlights the utility of dual-spray sources for rapid screening of reactivity and generating highly reactive compounds *in situ*.

Very recently, Lee *et al.* used time-resolved FD-MS in order to track fast reaction progress in fused microdroplets with microsecond time resolution, which is the current lower time limit for time-resolved MS reaction monitoring.^61^ They determined the timescales of their technique by monitoring droplet generation, fusion, and velocity using a high-speed camera. Aqueous droplets did not change in size during the analysis, and reactions were assumed to be quenched by evaporation upon entering the mass spectrometer. By varying the distance between the inlet of the mass spectrometer and the ion source, reactions could be monitored from approximately 15–50 μs. Using this technique, Lee *et al.* determined the microdroplet phase kinetics of phenolindophenol reduction by ascorbic acid, acid-induced cytochrome c unfolding, and H/D-exchange of bradykinin. Surprisingly, despite that droplet evaporation was not significant, the reduction of phenolindophenol was accelerated by approximately 1000-fold. This suggested that reaction rate enhancement in the microdroplets was perhaps due to confinement of the reagents within microdroplets. Subsequent work has shown that this reactivity enhancement can depend on several factors, including droplet charging.^13^

Solvent-assisted electrospray ionization (SAESI) is a recently developed variant of EESI. Instead of positioning the two sprayers so that the droplet plumes interact, the two sprayers are actually in contact and the two samples are believed to mix in a combined Taylor cone (*i.e.*, b in Fig. 4 is zero).^62^ This technique is functionally similar to EESI, and conceptually very similar to nanospray ionization from theta-glass capillary emitters or dual channel microchip emitters (*vide supra*), except that droplet lifetimes are likely longer as nanospray tends to generate smaller droplets that induce faster ionization. In reporting the development of the technique, Zhang *et al.* showed that SAESI-MS was able to efficiently ionize intermediates of a Au-catalyzed cycloisomerization of phenylpropargyl ether in the solvent DCM by co-spraying with MeOH. Similar experiments using EESI and ESI of DCM solutions diluted into MeOH contained more noise and artifacts. It was not clear why the SAESI spectra were cleaner. However, the authors suggested that SAESI avoids complex ion–molecule reactions and has one fewer parameter to be optimized than does EESI.

Miao *et al.* developed a very clever ESI variant for submillisecond time-resolved MS that involves two reagents and one ionizing flow.^63^ They combined reagents in a tee-mixer using an HPLC pump to achieve high flow rates in the tubing. The stream of liquid exiting the tee was directed to a secondary ESI source for ionization of reagents and any formed intermediates or products. The authors referred to their source as a DESI variant; however, they did not ionize or desorb compounds from a surface, so technique is most likely best described as ESI or SESI. By varying the distance between the T-mixer and the ion source, they achieved reaction times from 2.5–4.2 ms with submillisecond time resolution. As proof of concept, the investigators showed the reduction of phenolindophenol with ascorbic acid.

#### 2.1.2 Laser ionization techniques

##### 2.1.2.1 Matrix assisted laser desorption ionization and related techniques

Matrix-assisted laser desorption/ionization (MALDI) and related techniques operate in a vacuum and are inherently “offline” when studying reactions on anything but dry surfaces. Despite this limitation, ionization in MALDI has advantages relative to electrospray-based methods: (i) analytes are generally singly charged, which simplifies analysis for compounds prone to generating multiply charged ions; (ii) a variety of matrices exist with unique and differential ionization characteristics, and some analytes even ionize in the absence of matrix; and (iii) no potential is applied to the sample, limiting spurious electrochemistry during ionization. However, there are also significant disadvantages to MALDI and related laser ionization techniques: (i) small molecule matrix additives generally interfere with the data, making it difficult to observe compounds with molecular weights under approximately 500 g mol^–1^; (ii) sample handling generally occurs in air, which can lead to degradation of sensitive samples; and (iii) analytes with significant UV absorption profiles can undergo photolytic fragmentation during ionization. Furthermore, gas-phase charge transfer reactions between analytes and matrix molecules as well as photoelectrons present both a challenge for data interpretation and an opportunity for neutral analyte detection in laser ionization techniques.^64–67^

The characteristics of MALDI as a tool to ionize coordination and organometallic complexes has recently been reviewed.^68^ The applications of MALDI for offline analysis of a variety of small molecule and biological processes are legion, and these studies are too numerous to review here. Instead, we will focus on the development and specific use of MALDI as a tool to intercept reaction intermediates and study mechanism.

**Fig. 6:**
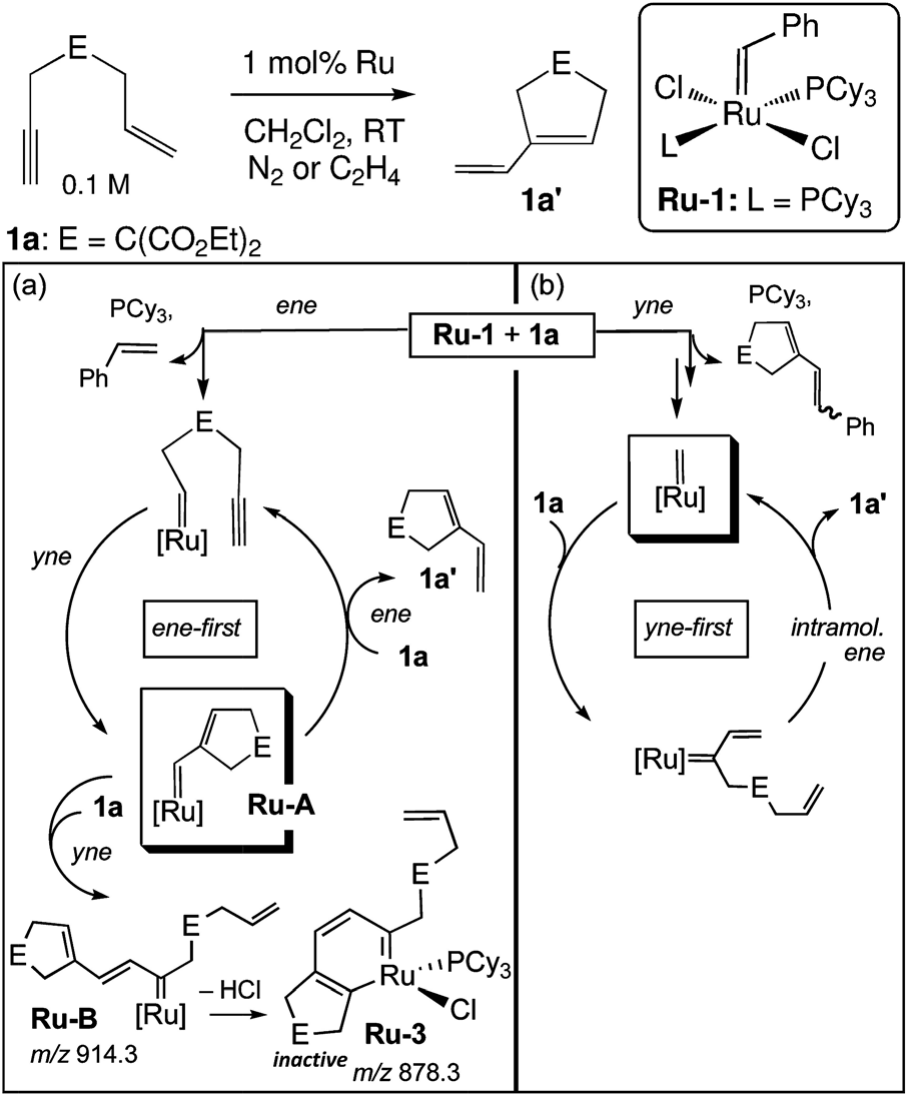
Mechanisms for (a) catalyst degradation and (b) productive metathesis during enyne metathesis as supported by MALDI-MS. Adapted with permission from ref. 70. Copyright 2011 American Chemical Society.

A significant disadvantage of MALDI as a tool to intercept reactive intermediates is that these compounds are not necessarily air-stable. Eelman *et al.* circumvented this issue by directly interfacing a MALDI mass spectrometer with a glove box.^69^ By relying on the aprotic and generally unreactive anthracene as a UV absorbent charge transfer matrix, they demonstrated that highly sensitive inorganic compounds such as Ti(iii) polymerization catalysts, Grubbs olefin metathesis catalysts, Rh–phosphine complexes, and Cu(ii) triflates could be observed with minimal decomposition. The investigators monitored the speciation and decomposition of Grubbs catalysts during enyne metathesis.^70^ Not only was an organometallic ruthenacycle identified as a decomposed Ru species in solution, but significant evidence was amassed that catalysis proceeded through an “yne-first” pathway, where the alkyne adds to the Ru carbene first and decomposition proceeds *via* a side-reaction of the “ene-first” path (Fig. 6). For some systems, a glovebox is not necessary, and ligand substitution at sensitive lanthanide metal centers has been studied by preparing MALDI analysis plates in inert-atmosphere glovebags.^71^

The single most important limitation of MALDI and related laser-based ionization techniques is that they are not readily interfaced with liquid reaction media because they generally require a vacuum to function. Some techniques have been developed to facilitate “online” MALDI analysis,^72^ but they have yet to be applied for direct monitoring of chemical processes in solution.

##### 2.1.2.2 Atmospheric pressure laser ionization techniques

The advent of laser ionization techniques that can operate at atmospheric pressure has significant and *largely unrealized* promise to bring MS to bear on unexplored classes of reactions and processes. Two very similar and heavily hyphenated laser ionization techniques have been used to monitor reactions in solution. The first was referred to as infrared matrix-assisted laser desorption electrospray ionization (IR-MALDESI).^73^ The technique is functionally very similar to AP-MALDI, where a solution of analyte is flowed to a capillary end placed in front of an MS inlet. A constant electrospray source is directed at the MS inlet, orthogonal to the analyte capillary. A focused IR laser (2.94 μm) ablates the solution at the end of the capillary and sends analyte into the electrospray plume. Ionization is believed to occur through the action of the orthogonal electrospray. The chelation of Fe(ii) by 1,10-phenanthroline, insulin denaturation, and cytochrome c digestion by trypsin were monitored. A similar technique known as electrospray-assisted laser desorption/ionization (ELDI) has also been applied for reaction monitoring.^74^ ELDI is very similar to IR-MALDESI, except that a UV laser (337 nm) is used for desorption/ionization, and the surface of a bulk analyte solution is directly analyzed instead of being flowed from a capillary. It is not entirely clear in this case whether ionization occurs through action of the laser or the orthogonal electrospray. Using this technique, olefin epoxidation, a variety of transition metal chelations, and protein digestion were monitored in real-time.^74^

While these examples demonstrate that online reaction monitoring is possible with laser-dependent ionization techniques, they have not made it clear whether there are advantages of atmospheric laser ionization over the much simpler electrospray methods. This is particularly true for the hyphenated techniques that rely on ESI for ionization. In order to fully realize the potential of online reaction monitoring with an atmospheric pressure laser ionization technique, processes that are induced by light absorption or involve compounds that are not readily ionized by electrospray-based methods could be explored. One example of monitoring a light induced reaction by mass spectrometry has recently appeared; however, this technique did not use the laser to induce ionization, but instead the illuminated microdroplets impacted on a charged steel needle in order to induce ionization.^75^

#### 2.1.3 Plasma ionization techniques

##### 2.1.3.1 Direct analysis in real time and related techniques

**Fig. 7:**
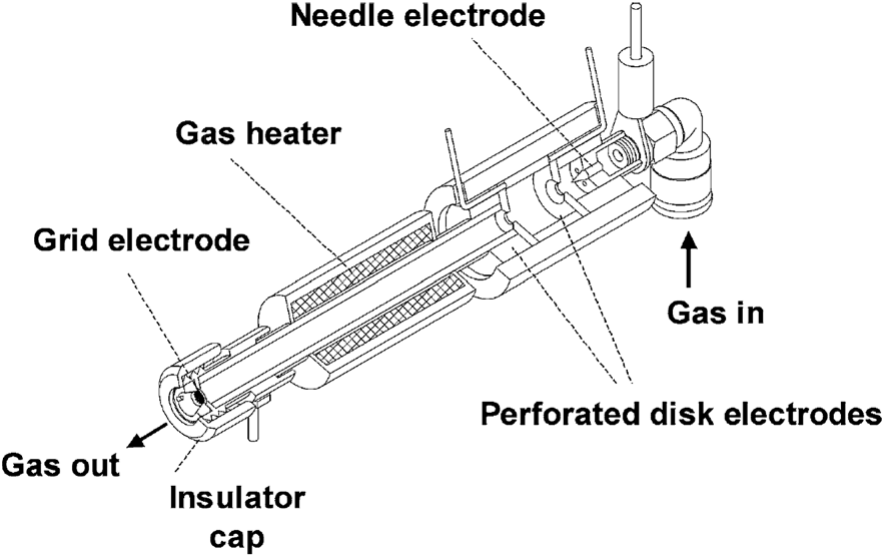
Cross sectional diagram of a DART source. Reprinted with permission from ref. 76. Copyright 2005 American Chemical Society.

Plasma ionization involves harsh conditions. However, it is possible to ionize molecules under ambient conditions that are difficult to observe using electrospray-based techniques. Therefore, plasma-based ionization techniques, most notably the ambiguously named direct analysis in real time (DART), have been applied to monitor a variety of reactions. The ionization source is based on a flow of heated and electronically or vibronically excited inert gas (generally He, Ar, or N_2_) incident on a surface or sample (Fig. 7). In air, these excited species interact with and ionize compounds such as water vapor which serve as proton donors and assist in ionization.^76^

DART has been used to monitor homogeneous reactions, where samples were taken from ongoing reactions and placed in front of the plasma source. Reagents and products of simple indole *N*-methylation and heterocycle debenzylation reactions were observed.^77^ The relative intensities in the DART mass spectra were in very good qualitative agreement with LC-UV data. DART was further validated as a broadly useful tool for preclinical pharmaceutical development, including reaction monitoring, in particular for rapid process optimization for the synthesis of compounds that are difficult to ionize by LC-MS.^78^ Safety hazards, such as the high temperatures and voltages, associated with DART make it unsuitable for direct monitoring of organic liquid surfaces. In part to address this problem, a low-temperature plasma probe was developed and applied^79^ to monitor liquid-phase reactions in real time. This technique has significant promise in the application of MS to study solution-phase processes involving molecules that are not well ionized by electrospray-based techniques.

As discussed below, the application of multiple orthogonal techniques is often rewarding in mechanistic science. In one very nice example, DART-MS was applied to cross validate an ESI-MS study on halogen bonding in solution. A gas-phase [M + I]^–^ adduct was detected by ESI-MS and DART-MS from solutions containing the analyte and iodide but was not observed in DART-MS where I_2_ was introduced into the ionizing gas stream.^80^ This result suggested that the formation of this adduct occurs in solution and not the gas phase.

### 2.2 Gas–liquid interface reactions

**Fig. 8:**
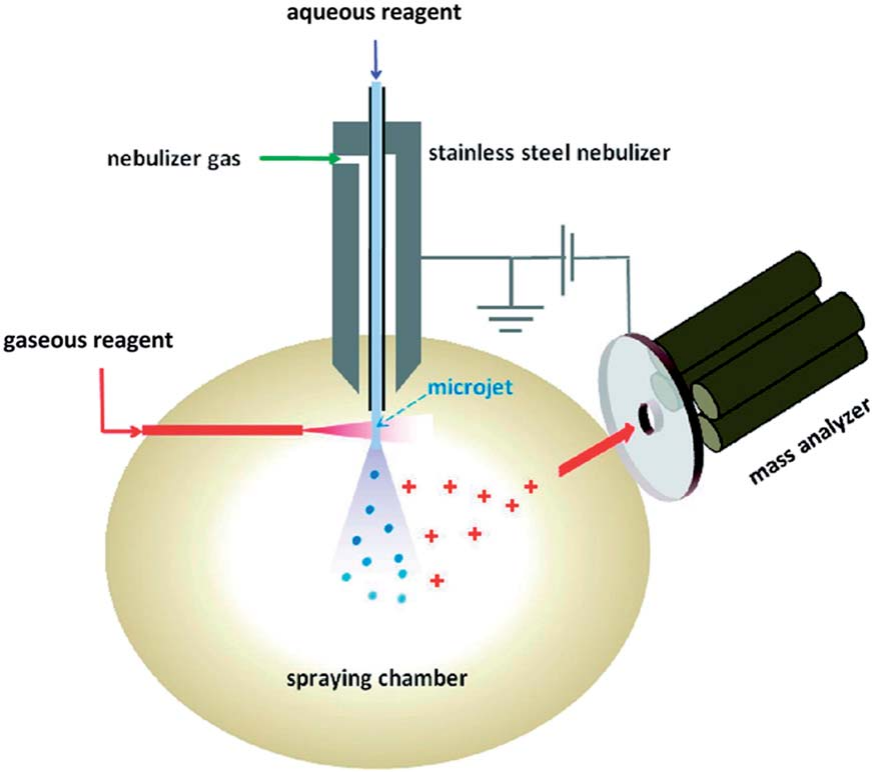
Online electrospray apparatus. Adapted with permission from ref. 90. Copyright 2010 American Chemical Society.

Reactions occurring at gas–liquid interfaces are important to atmospheric chemistry, environmental health, and aerosol science. Furthermore, it is a significant analytical challenge to specifically observe reactions as they occur on liquid surfaces. In terms of mass spectrometric monitoring of these processes, a few electrospray-based methods have been developed to explore reactions and dynamics at gas/liquid interfaces. Ionization from charged droplets is specific to analytes at the surface,^81^ which suggests that electrospray-based methods are particularly suitable for monitoring interfacial chemistry. The Colussi lab (Caltech) designed^82^ a variant of SESI where sample solutions are pumped through a grounded spray-source and nebulized using a high velocity coaxial gas flow, similar to SSI.^39^ From this stage, droplets evaporate and ions are released in a manner similar to electrospray ionization. To study reactions, reagent gas flows of various concentrations are introduced in the spray chamber, perpendicular to the nebulizer, and oriented in a way so that the gas interacts either with the plume of droplets or with the jet of liquid at the incipient plume (*i.e.*, before the sheer forces of the co-axial gas flow nebulizes the liquid stream) (see Fig. 8). Depending on the setup, Enami *et al.* estimated that the liquid surfaces interacted with the reagent gases for a minimum of a few microseconds^83^ to up to a millisecond.^82^ Droplets enter the mass spectrometer and ions are liberated within a few milliseconds of reaction initiation. The Colussi lab generally verifies the surface specific nature of their setup by introducing the reagents into the bulk spray, where the products found while introducing gaseous reagents are different or not observed. Gas phase reactions are more difficult to rule out, except that the concentrations of reactive gases in the spraying chamber are very low. These conditions not only allow for a selective probing of interfacial processes but give access to microsecond reaction timescales and can detect intermediates within milliseconds. The speed of the analysis limits secondary reactions and allows for the detection of otherwise unstable intermediates. Enami *et al.* have referred to this method as online thermospray MS.^84^ Using this technique, they have studied the interfacial reactivity of a variety of environmentally relevant compounds as well as the unique dynamics of proton transfer at the air/water interface.

In the initial application of this technique,^82^ Enami *et al.* studied the interfacial oxidation of I_aq_^–^ by O_3_(g), which generated IO_3_^–^ and I_3_^–^ as products. They discovered that interfacial I^–^ can catalyze the oxidation of Cl^–^ and Br^–^ by O_3_(g), yielding precursors of elemental chlorine and bromine which were not formed in the absence of I^–^. These results suggested that fine sea aerosols could provide a source of atmospheric bromine, a powerful ozone depleter.

Enami *et al.* have used their technique to study from the perspective of environmental health the interfacial reactions of antioxidants with O_3_(g), focusing on the pH-dependence of co-oxidant mixtures. They examined the interfacial ozonolyses of components of epithelial lining fluids present on the surface of lung tissues: ascorbic acid,^85^ uric acid,^85^ glutathione,^86^ and α-tocopherol.^86^ In the case of ascorbic acid, they found that it efficiently absorbed the oxidizing power of O_3_(g) to form harmless mixtures of dehydroascorbic acid and threonate at physiological, near-neutral pH. Under pH 5, however, interfacial ascorbic acid was converted to persistent oxygenated species proposed to be ascorbate ozonides which conceivably could cause oxidative damage to tissues.^86^ The studies highlighted some key differences between bulk solutions and aqueous surfaces: (1) relative rates of reactions with ozone between compounds were different at the surface *vs.* the bulk; and (2) different reaction products can be formed at air/water interfaces than in bulk solutions, because the ozonide and O-atom transfer products detected for uric and ascorbic acid and glutathione were not present during oxidations in bulk solution with O_3_(aq). These studies present a detailed picture of the divergent and pH-dependent reactivity of anti-oxidants at air/water surfaces. This unique picture would be difficult to obtain by other means.

The Colussi lab (Caltech) has used their method to study the oxidation of organic molecules in aerosols induced by ozone or hydroxyl radicals. In the ozonolysis of β-caryophyllene in 4 : 1 MeCN : H_2_O aerosols, carboxylic acid products were formed within milliseconds after microsecond exposures to O_3_(g). The rates of these reactions and distributions of products could not be explained by either gas- or liquid-phase oxidations. The Colussi group suggested that ozonolysis proceeded by a mechanism unique to the interface.^87^

Very recently Enami *et al.* studied Fenton chemistry occurring at the surface of aerosol droplets, and found that the kinetics and dynamics of this process are fundamentally altered at aqueous surfaces.^88^ Exposing solutions of FeCl_2_ to O_3_(g) or H_2_O_2_(g) led to the oxidation of Fe(ii) to Fe(iii) and the formation of putative Fe(iv)=O oxo species.^88^ The relative ratios of these products were highly dependent on the type of oxidant, O_3_ or H_2_O_2_, oxidant concentration, the solution pH, and the concentration of Fe. Adding small concentrations of DMSO, an O-atom acceptor, significantly lowered the intensity of a dimeric oxo species, while the monomeric compound, Fe(iv)OCl_3_^–^, was unaffected. This suggested that, at least at the air/water interface, the dimeric oxo compounds are more active for O-atom transfer than the monomeric compound. Furthermore, the estimated rates of Fe(ii) oxidation by either O_3_ or H_2_O_2_ were several orders of magnitude larger at the liquid surface than in bulk solution. The authors suggested that Fe(ii) ligand dynamics or coordination structure are significantly impacted by being at the air/water interface.

Some of the more intriguing studies that have been performed using online thermospray ionization have investigated the fundamental thermochemistry and dynamics of proton transfer at air/water interfaces. Enami *et al.* have studied the protonation of NMe_3_(g),^89^ hexanoic acid,^90^ and terpenes^83^ at the surface of acidic (*i.e.*, pH < 4) droplets. In general, each of these compounds were protonated when the pH of the bulk solution was approximately 3.5, suggesting that availability of protons at the air/water interface was being measured rather than gas-phase basicity.^91^ In the case of terpenes, this protonation surprisingly induced cationic oligomerization of these compounds despite the presence of water, which generally reacts quickly with carbocations.^83^ Furthermore, gaseous hexanoic acid was deprotonated by droplets whose bulk pH was greater than 2, suggesting that hydroxide anions were present at the air/water interface above pH 2.^92^ These studies strongly support the notion that the “neutral” pH for interfacial water is approximately 3 and that the H_3_O^+^ and ^–^OH present are of an acidity and basicity that lies somewhere between that of the solvated and gaseous ions.^91^ This work has challenged the simple notion that the surfaces of microdroplets are “acidic”.^91^ The data present compelling evidence that autoionization is more prevalent at aqueous interfaces and that the partially solvated H_3_O^+^ and ^–^OH that form are incredibly reactive. Colussi and Enami have neatly summarized this work in a comment on one of their original publications on this topic.^91^

**Fig. 9:**
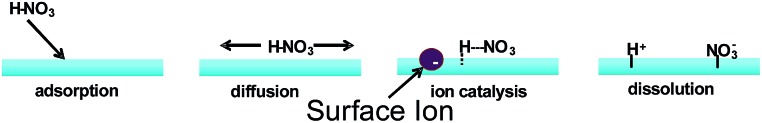
Scheme for general anion-catalyzed dissociation of HNO_3_(g) on water surfaces.

Very interestingly, Mishra *et al.* found using this technique that proton transfer from gas-phase nitric acid to interfacial water does not readily occur for neutral solutions.^93^ HNO_3_(g) dissociates on the water surface only in the presence of at least 1 mM electrolyte, as detected by the appearance of NO_3_^–^ and protonated hexanoic acid, C_5_H_11_COOH_2_^+^, in the mass spectrometer. On the basis of these experiments and a theoretical investigation, Mishra *et al.* proposed that HNO_3_(g) adsorbs onto the air/water interface to form HNO_3_(aq), but does not dissociate due to a lack of solvent stabilization of the incipient NO_3_^–^. In a process similar to heterogeneous catalysis, HNO_3_(aq) is proposed to diffuse along the surface until it encounters an anion site (Fig. 9). The presence of a negative charge is believed to electrostatically stabilize the proton transfer to the surface.^93^ A similar process is proposed to occur during the hydrolytic adsorption of NO_2_(g) into HONO(g) and HNO_3_(aq) on liquid surfaces.^84^

#### Field-induced droplet ionization

Grimm and Beauchamp developed field-induced droplet ionization in 2003.^94^ The technique functions by applying large electric fields to neutral droplets in order to induce polarization and ESI from both poles of the droplet. It was extended to hanging droplets of analyte from a silica capillary in order to study the liquid–gas interfacial reactions on the droplet surfaces after exposing the droplets to reactive gases.^95^ Gaseous naphthalene adsorption into droplets as well as ozonolysis reactions of unsaturated fatty acids were observed with 10–60 second reaction times. Subsequent studies examined the interfacial reactivity of surfactant proteins,^96^ pulmonary phospholipids,^97^ and cholesterol sulfate.^98^ In each case, the dynamics of interfacial ozonolysis during droplet exposure were different than in bulk solution, suggesting the importance of a unique environment at the liquid surface.

## 3. Relevancy

The measurement of intermediates during ongoing chemical reactions in the condensed phase by *in situ* spectroscopy is plagued by the problem of not knowing if the detected compounds are relevant to the overall chemistry. This issue tends to become especially complicated during spectroscopic analysis of catalysis, as the prevalent species need not significantly contribute to the observed reactivity. The conundrum is that if a compound is not observed, it may still be present, and if a compound is observed, it is not clear what its significance is. Halpern's classic study of enantioselective olefin hydrogenation with chiral (bis-phosphine)rhodium complexes is a perfect example of this situation: the most stable and abundant intermediate gave rise to the minor product of catalysis.^99^ MS is particularly afflicted by the fact that the detection generally occurs in the gas phase, which requires a series of processes to ionize compounds and transfer them out of their native environment. These processes introduce doubts as to whether the data are free of artifacts and representative of the system under study. In particular, signal is not generally directly proportional to concentration in solution, and different ionization techniques can yield drastically different responses for different classes of analytes. MS may not lie, but it can mislead.

For these reasons, mass spectrometric data of ongoing reactions in the condensed phase always should be viewed skeptically before deciding what they mean. ESI-MS studies can generally rely on a relatively well understood ionization mechanism while examining results. This luxury does not necessarily exist for reaction studies with novel ambient ionization methods. Often times, a significant number of control experiments are required before an experiment can be properly interpreted. In this section, we examine, with examples from the literature, a series of questions which we believe should be addressed when gathering data from a MS study. Not all of the questions need to be answered in order for useful information to be obtained. In fact, it may be impossible to address each of these points for some systems. Instead, they are meant to serve as a guide for deciding precisely what a given set of data does or *does not* mean, and which experiments could be most illuminating. For most chemical processes, it will be evident that the judicious application of multiple and orthogonal techniques is an efficient route to finding answers.

### 3.1 When and where is an ion or compound produced?

For a MS study, knowing where a compound is formed is generally required for any meaningful interpretation. Electrospray-based ionization methods are plagued by spurious redox processes that can occur during ionization to generate artifacts.^38^ This has been noted for DESI in particular.^100,101^ The presence of electrochemistry or oxidation can generally be assessed by a series of control experiments that vary the ionizing potential, the presence of oxygen, the solution conductivity (additional electrolyte), the analyte concentration, and flow rate. Memory effects from previous samples are also a common problem in MS. “In-source” fragmentation of gas-phase ions at a mass spectrometer inlet can occur before they reach the high vacuum portion of the instrument. This gives rise to nonrepresentative species but they can be readily tested for by studying the effect of the voltage and temperature settings near the front end of the mass spectrometer.^102^ Isotope labeling can also be particularly powerful to discern the origin of a species, often with the simultaneous confirmation of composition and structure. These simple experiments should be performed for any study meant to investigate chemical mechanisms (as opposed to simply track reaction progress).

An additional, and very difficult, aspect of this question is determining when compounds form relative to when the experimenter wants them to be. In other words: Is a given intermediate formed in solution or purely as a result of the analysis? The chemical environment of a microdroplet is unique, and differs depending on the overall charge of the droplet.^13,61^ It is not uncommon for reactions to have enhanced rates while molecules are in droplets, or for otherwise impossible processes to occur.^103,104^ Therefore it is important to distinguish whether certain compounds are observed due to reactions that occurred in the bulk solution, or while droplets were in flight. In the case of reactive DESI, EESI and similar techniques, this question is complicated by the fact that the reaction is initiated *during* analysis.

It is important to understand the timescale of electrospray processes in order to address this point. Under ambient conditions, ionization from larger microdroplets, such as those generated by DESI, *tends* to be on the order of milliseconds. Analytes in nanodroplets, such as those generally produced during nano-electrospray (nanospray), can enter the gas phase within microseconds.^81^ Also, we have seen that, once droplets enter a heated mass spectrometer inlet capillary, evaporation and ionization can be very fast and on the order of microseconds even for DESI like processes.^37,61^ Therefore, once gas-phase processes have been ruled out, whatever chemistry that can occur must be very rapid to significantly impact results.^105^

Furthermore, gathering data from two unique, but compatible, ionization techniques with differing ionization dynamics can be insightful. We have utilized both ESI and nanospray to study Pd-catalyzed aerobic oxidations and found that the data were identical from both ionization techniques, even though the timescales of nanospray ionization are orders of magnitude different than that of electrospray.^106^ As such, the observed Pd species most likely formed in solution prior to ionization. It is worth pointing out that the timescale argument laid out above assumes that reactions in droplets are similar to the bulk phase, which has been shown to be false for a wide range of reactions.^13,61,88^ Thus, this argument only applies when *in droplet* reactivity is slow relative to evaporation and ionization.

The most common way in practice to probe when ions form is to determine how relative MS intensities behave as the reaction time is systematically varied. Quantities of reagents tend to decrease with time while amounts of intermediates and products rise. Given enough reaction time, the intensities of intermediates are expected to eventually fall. With some techniques, time is directly variable, such as during a minutes- to hours-long reaction being studied by ESI-MS. This is more complicated for fast reactions and analysis methods where initiation occurs during ionization, such as DESI- or EESI/SESI/FD-MS. Often, the only way to vary reaction “time” is to increase the distance between the initiation site and the MS inlet, which could affect other aspects of the ionization process. This has been shown to give reasonable data for dual-spray techniques.^61^ However, some ionization techniques, such as DESI, are very sensitive to geometry parameters and do not readily allow for such variation. If ion abundance does not vary as expected with reaction time, then the model needs to be revisited: either the suspected role of a compound is not correct or the species is not predominantly formed or broken down during the process that is being varied (*e.g.*, droplet flight time). Observing this phenomenon does not necessarily rule out the presence of the species prior to ionization, but it does indicate that alternative formation mechanisms should be explored. Also, because it is difficult to directly relate MS intensity to concentration, this kind of data should be regarded as qualitative and non-determinative.

Systematic and independent variation of both reaction and ionization timescales typically provides compelling evidence regarding the origin of observed species. The numerous tunable ionization methods available (*vide supra*) makes addressing this question reasonable to approach for most systems. For some techniques, however, this question remains difficult to address. In these cases, control experiments with other more readily variable ionization techniques can be used to cross-validate data.

### 3.2 What is the structure of the detected compound?

The boon and bane of reaction monitoring by MS is that it is not an information-rich technique. Each ion in the gas phase is collapsed to a single datum: an *m*/*z* value. This is immensely helpful in sorting out the generally complex speciation problem that is a chemical reaction but the amount of structural information for each compound is very limited to nonexistent. Intramolecular processes, enantioselective reactions and isomerizations are particularly difficult to monitor by MS, because each compound has the same *m*/*z*. Independent synthesis and structural characterization by X-ray diffraction is the gold standard for structure determination, but it is a laborious and difficult process that is only worthwhile in a few instances. Furthermore, some catalytic and reaction intermediates are not easy to isolate.

Fragmentation by MS/MS is a traditional method for structural characterization of gas-phase ions; however, this technique is crude and does not generally discern between structurally related compounds, such as those on a catalytic cycle. In the case of metal complexes, in our experience, fragmentation of the unchanging supporting ligand is a common occurrence that limits detailed structural analysis by MS/MS.^35^ This is in contrast to the wide use of MS/MS as a structural tool in bioanalytical chemistry, where libraries of peptide, lipid, and protein fragmentation spectra can be used to elucidate the primary structure of common compounds. The most significant advantage of MS/MS is that it is easy to perform: it is available with most commercial instruments and generally accomplished by the click of a button. Therefore, as MS/MS can sometimes yield helpful information, it is commonly applied in attempts to make structural assignments.

Marquez *et al.*'s study of l-prolineamide catalyzed α-halogenation mentioned above is a good example of how data obtained by MS/MS can shed some light on an isomerism problem.^58^ The authors used MS/MS to distinguish between the C–X species (X = Cl, Br, I), putatively detected by ESI, and the N–X species, putatively detected by EESI (Fig. 5). During a chlorination reaction initiated during EESI, the MS/MS spectrum of *m*/*z* 203 showed a fragment at *m*/*z* 104 that corresponded to a molecular formula of C_4_H_7_NCl^+^. This peak was not present during ESI-MS/MS studies of the same system. The data provide conclusive evidence that the composition of the ion packet at *m*/*z* 203 differs between EESI and ESI, but there is little specific structural information. Molecular assignments are then based on supposition. This case is general for most systems where detailed studies of the gas-phase fragmentation of the same or similar compounds are not available or readily accessible.

**Fig. 10:**
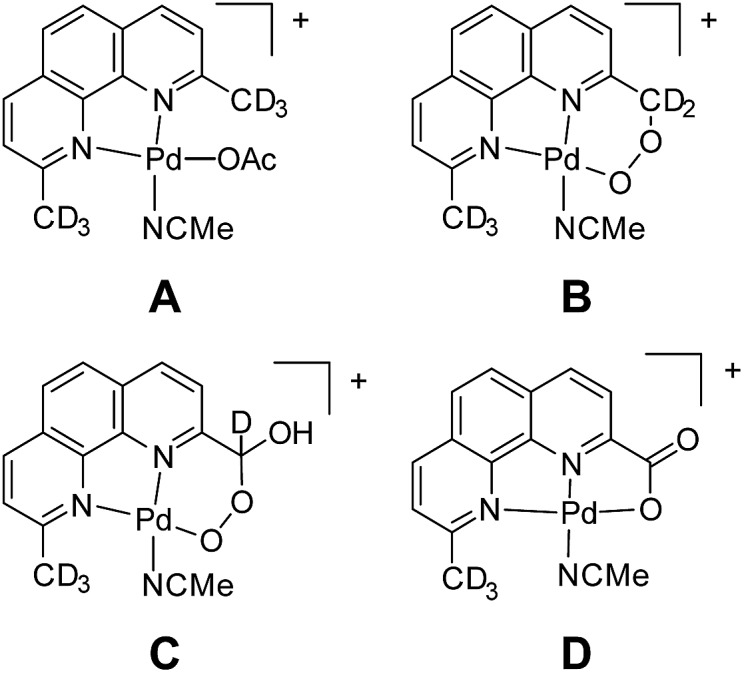
Structural elucidation of oxidized ligand species *via* isotope labelling.

In our experience, judicious isotope labelling studies are a generally reliable and informative means to confirm composition and progeny of ions. In many cases, there are only a few reasonable isomers for a given *m*/*z*, especially when high-resolution data are available. Intermediates involved in functionalization, substitution, and elimination reactions are often discernible by isotope labelling at specific locations. Unfortunately, this method is limited by synthetic access to specifically labelled precursors. In a case from our own research, isotope labelling was used to elucidate the primary structure of ions corresponding to an oxidatively degraded aerobic Pd alcohol oxidation catalyst (Fig. 10).^107^ Labelling with ^18^O_2_ confirmed the aerobic origin of the oxygen atoms in the oxidized compounds. To discern the methyl functionalization of each compound, the –CD_3_ variant of the complex, **A**, was prepared and its degradation products **B**, **C**, and **D** were analyzed by MS. The sequential loss of deuterium conclusively demonstrated the substitution of each compound. Prior to this study, the penultimate peroxyl acetal **C** was particularly difficult to assign. Furthermore, this selective labeling experiment simultaneously confirmed that the methyl groups of other Pd species were intact.

Ion spectroscopy is a promising technique for electronic and structural characterization of ions in the gas phase. Obtaining spectroscopic information of ions generally involves specialized and custom equipment but a number of approaches exist for obtaining gas-phase UV-vis and IR data.^17,18^ The vast majority of techniques are based on photolytic dissociation or fragmentation of a mass-selected ion with subsequent detection by MS. The absorbance of photons is measured by the decrease in intensity of the mass-selected parent ion as a function of excitation wavelength.

One elegant method for obtaining IR data of gas-phase ions is known as cryogenic ion vibration predissociation spectroscopy (CIVP).^18^ A beam of ions is passed into a ∼10 K cryogenic ion trap, where they form weak association complexes with inert buffer gas molecules (*e.g.*, N_2_, Ar, H_2_). An ion adduct of interest is mass-selected and then passed through a tunable IR laser. When the tagged ion is in resonance with the IR beam, the weakly bound gas molecules dissociate to yield a lower mass ion. The ratio of tagged and untagged ions in the mass spectrometer is proportional to the IR absorbance of the tagged parent at that wavelength. An incredibly powerful aspect of this spectroscopic approach is that when two independent IR sources are used, isomer-selective spectra can be obtained.^18^ Thus, gas-phase spectroscopy has the potential to resolve questions of isomerism during MS studies.

**Fig. 11:**
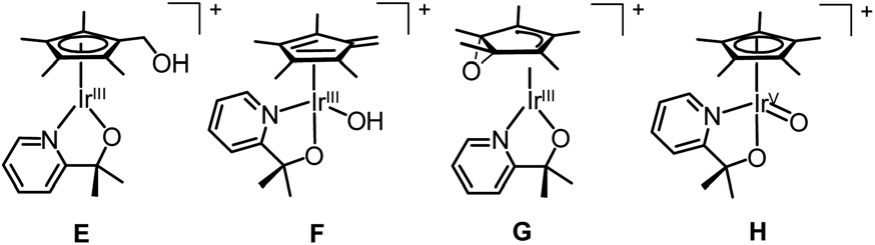
Potential isomers of oxidized Cp*Ir precatalyst.

We turned to CIVP to discriminate between the various isomers of an oxidized organometallic Cp*Ir compound that was a water oxidation precursor (Fig. 11).^35^ DESI-, SSI-, and ESI-MS of reaction mixtures revealed the presence of an initial precatalyst oxidation product. The structure of this compound was necessary to discern the initial mechanism of activation. Isotope labelling of the bidentate N, O ligand showed that it was not functionalized. However, we could not readily determine the Cp* functionalization by isotope labelling, because deuterium-labelled Cp* ligands are synthetically challenging to prepare. Gas-phase IR spectroscopy of ions cryogenically tagged with N_2_ revealed prominent O–H stretches, which ruled out the presence of compounds **G** and **H**. Detailed analysis of the Cp* stretches and comparison to DFT calculations indicated that the Cp* core was intact. This finding ruled out the fulvene, **F**, suggesting that this compound had a hydroxylated Cp* methyl group as in **E**. Furthermore, there was some secondary structural information where two rotamers of the Cp* ring of **E** were detected. Also, when CIVP spectra were recorded for further products of Cp*Ir oxidation, multiple constitutional isomers of Cp* hydroxylation were detected and characterized in the gas phase. Such detailed structural characterization of ions is uncommon, but can be highly informative for compounds that cannot be observed or isolated by techniques other than MS.

It is not reasonable to expect detailed structural analysis of every species detected during a given study. However, to realize the power of MS to observe and characterize ions, important compounds should be completely characterized with the relevant available techniques, including those orthogonal to MS. These studies have the capability to provide a unique perspective on intermediates that is otherwise inaccessible. Their expanded application to studies of reactive intermediates has the potential to significantly enhance the impact and acceptance of MS studies in the broader synthetic chemistry community.

### 3.3 What role does the compound play during the reaction or catalysis?

This question is the *raison d'être* of *in situ* reaction monitoring and is not unique to analysis by MS. In cases where a novel or unexpected intermediate is discovered, addressing this question is essential. Discerning or supporting a proposed role for a detected compound or intermediate is an extremely difficult task. This discussion is further complicated during studies of catalysis, where the constant breakup and formation of catalytic intermediates confounds analysis. A solid understanding of the underlying chemistry is required, which necessitates a multidimensional approach incorporating conventional mechanistic experiments, such as kinetics, and isotope labelling and scrambling studies. Having synthetic access to an intermediate is invaluable because its intrinsic reactivity can be explored.

In terms of ways to access this information by MS, simultaneous reaction monitoring and modelling of MS data has been shown to be useful for quantitative modelling purposes^108^ as well as qualitative characterization.^7^ These kind of data should be viewed critically, however, as mass spectra of complicated mixtures are only quantitative under strictly controlled conditions. In terms of catalysis, a common question arises as to whether a given intermediate is on the catalytic path. In some cases, when the intrinsic dynamics of a compound are slow relative to catalysis, it is possible to approach this question.

**Fig. 12:**
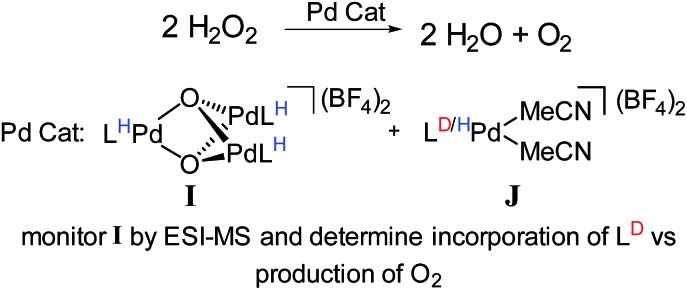
Isotope scrambling experiment devised to determine catalytic role of trinuclear Pd species.

For example, we examined the role of a trinuclear Pd_3_O_2_^2+^ intermediate, **I** (Fig. 12) that is present during aerobic oxidation and hydrogen peroxide disproportionation catalysis with Pd.^107^ Synthetic studies of this compound revealed that it was formed during catalysis and this process was not directly reversible (*i.e*., it is not in engaged in any dynamic equilibria). Therefore, we supposed that this compound was part of a dedicated catalytic path, but it was not clear whether its formation was the only catalytic path. Furthermore, the kinetics of hydrogen peroxide dissociation suggested the presence of multiple catalytic cycles.

To confirm the presence of multiple mechanisms and demonstrate the involvement of the trinuclear species, we devised an isotope scrambling experiment. We used ESI-MS to monitor the hydrogen peroxide disproportionation catalyzed by a mixture of perprotio **I** and a labelled co-catalyst [L*Pd(MeCN)_2_](BF_4_)_2_, **J** (Fig. 12). **I** itself does not directly exchange with [LPd(MeCN)_2_]^2+^, **J**, in solution, but intermediates in its synthesis statistically scramble with it. As expected, deuterium incorporated into the population of **I** during catalysis, which indicated that **I** was being simultaneously consumed and produced. By comparing the rate of deuterium incorporation to the rate of catalysis, we unambiguously showed that **I** is not on the sole path for catalysis. Incorporation was significantly slower than would be expected if breakup and formation of **I** were necessary for every molecule of O_2_ produced. Thus, this experiment discerned the presence of multiple simultaneous catalytic cycles. Similarly designed experiments also should have the capability to delineate whether species are on single catalytic paths or act as dynamic reservoirs of active species.

**Fig. 13:**
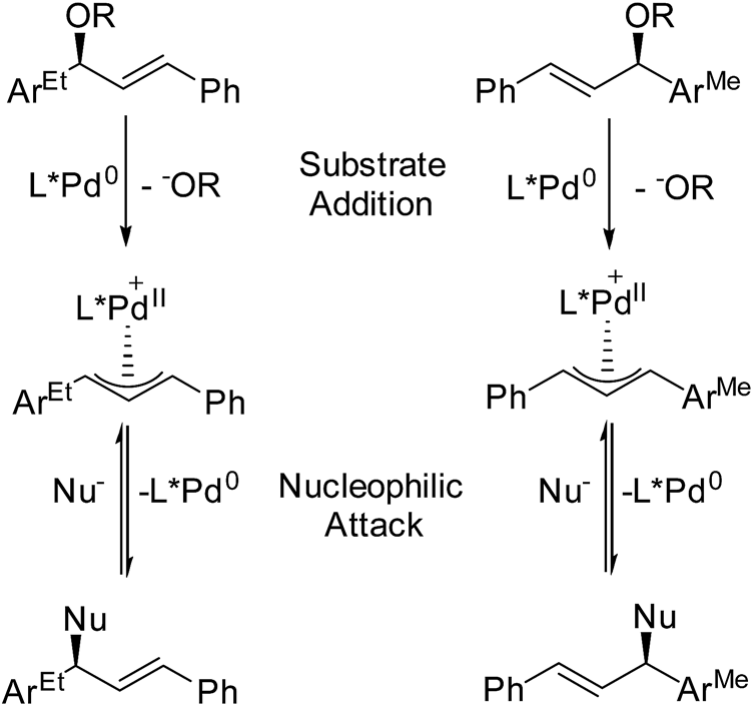
Enantioselective allylic substitution.

In terms of simultaneously resolving isomerism and roles during catalysis, the Pfaltz lab (University of Basel) has devised an ingenious technique for assessing how intermediates, which are detected during MS monitoring, contribute to enantioselectivity.^109^ By adding mass labels to sites that are distant from the reactive centers of enantiomeric compounds, they have created mixtures of “quasienantiomers” (Fig. 13). The investigators use equimolar mixtures of quasienantiomers as substrates with prospective chiral catalysts and use the relative intensities of the quasienantiomeric intermediates to predict enantioselectivity. They applied this method to a Pd-catalyzed allylic substitution reaction. In cases where the detected relative intensities agreed with preparative experiments, substrate addition was enantiodetermining.^110^ For these allylic substitutions, the overall reaction could be reversed, so the influence of nucleophilic attack on enantioselectivity could be independently examined. Reacting the Pd catalyst with a mixture of quasienantiomeric products yielded a mixture of Pd allyls whose relative intensities reflected the enantioselectivity of nucleophilic attack.^111^ Comparison of these results to preparative reactions provides a very simple way to differentiate enantiodetermining steps, a key question in chiral catalysis.

Ion–molecule reactions in the gas phase have been used to assess the fundamental, gas-phase reactivity of a suspected intermediate and confirm that a species is active for a particular transformation. As an example, Santos and Metzger have observed the polymerization of ethylene by cationic methyldicyclopentadienylzirconium(iv) (zirconocene) in the gas phase by introducing ethylene into the collision cell of a commercial mass spectrometer.^112^

## 4. Conclusions

MS provides a unique and powerful perspective in mechanistic investigations of reaction systems. However, like all techniques, it is seldom independently conclusive. The most informative studies tend to weave MS results in with conventional kinetics, synthesis, and isotope labelling experiments. The ability to access chemistry at short timescales with some electrospray-based ionization techniques has allowed chemists to support hypotheses, study otherwise undetectable reactive intermediates, and discover new mechanisms. Techniques not based on electrospray that rely on laser or plasma ionization have significant promise to bring mass spectrometry to bear on previously inaccessible systems, but these techniques are currently not well-explored. The recent explosion of techniques available to couple MS to ongoing reactions in most any media is expected to continue to provide specific insight into reactions mechanisms by detecting and exploring the chemistry of reactive intermediates *in situ*. Current technology grants us the extraordinary ability to target varied of reactivity by judicious choice or design of an ionization technique, accessing chemistry across three phases of matter and with microseconds to minutes of accessible reaction times. Instead of having to bring our reaction to the mass spectrometer, we can now bring the mass spectrometer to our reaction.
